# Basal thumb osteoarthritis surgery improves health state utility irrespective of technique: a study of UK Hand Registry data

**DOI:** 10.1177/1753193420909753

**Published:** 2020-03-12

**Authors:** Jennifer C. E. Lane, Jeremy N. Rodrigues, Dominic Furniss, Edward Burn, Robert Poulter, Matthew D. Gardiner

**Affiliations:** 1 Nuffield Department of Orthopaedics, Rheumatology and Musculoskeletal Sciences, University of Oxford, Oxford, UK; 2 Department of Plastic Surgery, Stoke Mandeville Hospital, Aylesbury, UK; 3 Royal Cornwall Hospital, Truro, UK; 4 Department of Plastic Surgery, Frimley Health Foundation NHS Trust, Wexham, UK

**Keywords:** Osteoarthritis, thumb, registries, surgical procedures, trapezium bone, trapeziectomy, patient reported outcome measures, Patient Outcome Assessment, Health Status

## Abstract

We used UK Hand Registry data to study two aspects of basal thumb osteoarthritis surgery: first, whether health-related quality of life improves after surgery. Second, whether results from trials comparing simple trapeziectomy and trapeziectomy with ligament reconstruction and tendon interposition are reproducible in routine clinical practice. Prospectively collected EQ5D index and Patient Evaluation Measure part 2 data were compared at baseline and at 3, 6, and 12 months postoperatively in 1456 patients (median age 67 years; 78% female). A mixed-effects regression model was also used to determine the postoperative trajectory of these variables. There was a significant improvement in the EQ5D index (median + 0.15; (interquartile range 0 to 0.40)) and Patient Evaluation Measure (–22; (–33 to –10)) by 1 year postoperatively and with no meaningful difference between the two techniques. This study demonstrates health state utility gains after basal thumb osteoarthritis surgery regardless of surgical techniques used.

**Level of evidence:** III

## Introduction

The choice of surgical method for treating base of thumb osteoarthritis (BTOA) remains controversial (Brunton and Wilgis, 2010; Deutch et al., 2018). Randomized control trials (RCTs) have shown little difference between procedures (Davis et al., 2004; De Smet et al., 2004; Field and Buchanan, 2007; Gangopadhyay et al., 2012; Wajon et al., 2015). These studies focus on comparing techniques of surgery for BTOA. They assume that there is real-world value from surgery for BTOA in general. Patient-reported outcome measures (PROMs), such as the EQ5D index, can be used to quantify the changes in health-related quality of life, which is also described as health state utility (Beard et al., 2018; Burn et al., 2018; Murray et al., 2014; Rombach et al., 2019). In BTOA surgery, there have been no national studies that have analysed preoperative health state utility, or used health state utility to compare surgical techniques (Efanov et al., 2019; Maru et al., 2012; Varitimidis et al., 2000; Yeoman et al., 2019).

Routinely collected data from everyday practice has greater generalizability of results than RCTs (Makady et al., 2018). For example, patients at the extremes of age and with greater levels of comorbidity are less likely to be included in RCTs, but are treated in practice and included in routine datasets. Thus, real-world data may provide a more realistic evaluation of an intervention, which may extend beyond the limitations of what is collected in a clinical trial setting (Garrison et al., 2007; Katkade et al., 2018). Patient registries have been used in other areas of musculoskeletal surgery, collecting PROMs and other outcome measures, to enable evaluation of techniques used in routine clinical care (ASPS, 2019; BOA, 2019; Hume et al., 2013)*.*

The UK Hand Registry (UKHR) was established in 2011 to align with other prospective national registries in musculoskeletal surgery (BSSH, 2019). The primary aim of this study was to assess change in PROMs after BTOA surgery. The secondary aim of the study was to compare patient reported outcomes after simple trapeziectomy and trapeziectomy with ligament reconstruction and tendon interposition (LRTI) in real-world observational data.

## Methods

### Study design

This cohort study used the UKHR, including all consecutive patients from 1 February 2012 to 31 January 2018. Exemption from ethical approval was confirmed by University of Oxford Clinical Trials and Research Governance prior to commencement of the study.

### Patients

Adult patients undergoing elective surgery for BTOA were prospectively invited to be included in the UKHR. Full written consent was provided by each patient before inclusion into the registry, and this research study is based upon secondary use of these data. Patients were limited to those under the care of a surgeon actively participating in the UKHR.

### Intervention

All patients underwent surgery as chosen in conjunction with their operating surgeon. In order to make the results comparable with previous clinical trials, we compared the two most commonly undertaken techniques – simple trapeziectomy or trapeziectomy with LRTI. Operative details were uploaded to the UKHR online platform at the time of surgery (https://www.ukhr.net). Identifiable data were anonymized prior to release from the registry for analysis.

### Clinical outcomes

Two PROMs were chosen to evaluate quality of life and hand-specific function after surgery. The Patient Evaluation Measure (PEM) was used to determine the impact of surgery on hand function (Macey et al., 1995). The 10-question section 2 of the PEM was used as originally designed. The 5-level EQ5D index was used as a generic score representing global quality of life to enable comparison with other medical interventions (Brazier et al., 1993; EuroQol, 1990).

Patients added to the registry were asked to complete PROMs at baseline prior to surgery and were then contacted remotely at 3, 6, and 12 months postoperatively to complete further PROMs using mail, email, or SMS messaging. Results were collated by a central administrator who was independent of the operating surgeons. All data were anonymized prior to analysis.

### Data analysis

Item-level data for each procedure at each time point were collected for both PROMs. Item-level data were added together to give a total score for the PEM (range of possible scores 10–70), and the EQ5D index score was calculated using the UK utility index for each timepoint using the EQ5D crosswalk value sets (range of EQ5D index –0.594 to 1.0 using English value set) (EuroQol, 2019). The total PEM and EQ5D index scores were then used to calculate the change between baseline score and each postoperative timepoint (3, 6, and 12 months postoperatively) for each patient, to produce a ‘delta’ score – an individualized change in PROM for each patient for each time point. These delta scores were used to determine an individual’s change in function. These were then combined to calculate a median change in pre and postoperative function of patients for the two surgical groups and to investigate the difference between the median change in function between the two surgical groups over the postoperative follow-up points.

Secondary regression analysis was undertaken to investigate the longitudinal trend in PROMs over the three postoperative time points to assess the trajectory of scores over time. This model was estimated for the subgroup of patients who had fully completed baseline and one fully completed postoperative questionnaire for both the PEM and EQ5D.

### Statistical methods

Kruskal–Wallis and Mann–Whitney *U* tests were undertaken to determine statistical significance between surgical groups. These were performed after histogram analysis of data distribution and Kolmogorov–Smirnov testing rejected the data having a normal distribution. Delta changes in PROMs postoperatively were only calculated where data were available undertaking available-case analysis (pairwise deletion), and missing data were not imputed.

Secondary analysis of the postoperative trajectory of both the PEM part 2 and EQ5D index used mixed-effects regression modelling to determine the effect of repeated postoperative time points upon PROM results for each individual. This iterative method does not impute missing values but estimates postoperative PROMs based upon data being missing at random for the other time points where data have not been completed.

After examining the relationship between continuous explanatory variables (age, baseline PROM score) and postoperative PROM score, the interaction between time and PROM score was tested to feed into the final model. There was little evidence of non-linearity between continuous explanatory variables and postoperative PEM part 2, and so a linear relationship was assumed. However, evidence of non-linearity was seen for age and preoperative score with postoperative EQ5D, and this was accounted for by using cubic splines. No interaction between time since surgery or either postoperative PROM score was found. For the final regression model, baseline EQ5D index and PEM part 2 scores were treated as continuous variables and mixed-effects linear regressions were undertaken to determine the impact of age, sex, baseline PROM score, and surgical treatment subtype on postoperative PROM scores. Where appropriate, Bonferroni-adjusted *p* value thresholds are provided to aid the interpretation of the results.

## Results

### Demographics

Over the 6-year inclusion period, 1456 patients were added to the UKHR, of which 749 underwent trapeziectomy alone and 648 underwent trapeziectomy with LRTI (Supplementary Figure 1). After the first year, recruitment of patients to the registry was steady during the study period. At baseline, the age, sex, and PROM scores of patients undergoing the two procedures were evenly matched (Table 1). There was attrition of the number of patients completing postoperative PROMs (Supplementary Figure 1). In order to evaluate the potential risk of bias from loss to follow up, the cohort of patients with and without follow-up were compared at baseline. Baseline demographic characteristics and PROMs were similar between those with and without follow-up, and they were also similar between patients in the two treatment groups (Supplementary Table 1).

**Table 1.** Baseline characteristics of trapeziectomy and trapeziectomy with LRTI groups.

Characteristics	Trapeziectomy (all patients)	Trapeziectomy with LRTI (all patients)
Median age, years (IQR)	67 (60 to 72)	66 (59 to 71)
Female sex	77%	78%
Median baseline EQ5D index (IQR)	0.69 (0.26 to 0.80)	0.66 (0.26 to 0.78)
Median baseline PEM part 2 score (IQR)	49 (41 to 56)	49 (40 to 55)

LRTI: ligament reconstruction and tendon interposition; PEM: Patient Evaluation Measure.

### EQ5D index

There was a significant improvement in the EQ5D index following surgery across the whole study population (*p* < 0.01; Table 2). When comparing the difference between trapeziectomy or trapeziectomy with LRTI, no significant difference was found at 3 months (*p* = 0.20) and 12 months (*p* = 0.57), with a non-clinically meaningful difference in the EQ5D index at 6 months (median difference between trapeziectomy and trapeziectomy with LRTI at 6 months: 0.05, *p* = 0.04; non-significant when Bonferroni-adjusted *p*-value threshold for significance is accepted: *p* = 0.017).

**Table 2.** Change in EQ5D index and PEM part 2 score postoperatively.

Patients	EQ5D index			PEM part 2		
	3 months	6 months	1 year	3 months	6 months	1 year
All patients	0.07 (–0.02 to 0.25)	0.10 (0 to 0.29)	0.15 (0 to 0.40)	–18 (–29 to –6)	–22 (–31 to –10)	–22 (–33 to –10)
Trapeziectomy	0.07 (–0.04 to 0.24)	0.08 (–0.04 to 0.24)	0.16 (0.004 to 0.42)	–18 (–29 to –6)	–21 (–31 to –9)	–22 (–33 to –10)
Trapeziectomy with LRTI	0.06 (0 to 0.28)	0.13 (0 to 0.34)	0.14 (0 to 0.37)	–18 (–29 to –7)	–23 (–31 to –10)	–23 (–32 to –10)

PEM: Patient Evaluation Measure; LRTI: ligament reconstruction and tendon interposition.

All data are presented as median score changes (IQR).

### PEM Part 2

There was a significant improvement in the PEM part 2 following surgery across the whole study population (Table 2; *p* < 0.01). There was no difference seen in reported functional improvement between those who underwent trapeziectomy or trapeziectomy with LRTI at any time postoperative point (3 months *p* = 0.93; 6 months *p* = 0.842; 1 year *p* = 0.97).

### Mixed effect regression analysis

**Figure 1.**
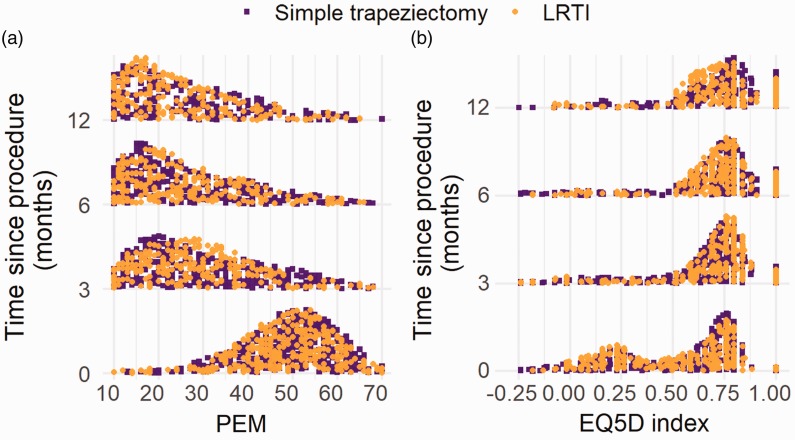
(a) Histogram of PEM part 2 score at baseline, 3 and 6 months, and 1 year postoperatively for those included in regression analysis. (b) Histogram of EQ5D index score at baseline, 3 and 6 months, and 1 year postoperatively for those included in regression analysis.

In the secondary analysis only including patients with fully completed PROMs at baseline and at least one postoperative time point, 746 patients were included. This subgroup had a very similar demographic profile to the full patient cohort (Supplementary Table 2). The change in the overall PEM part 2 and EQ5D index over the postoperative periods shows the overall improvement in scores (Figure 1).

**Figure 2.**
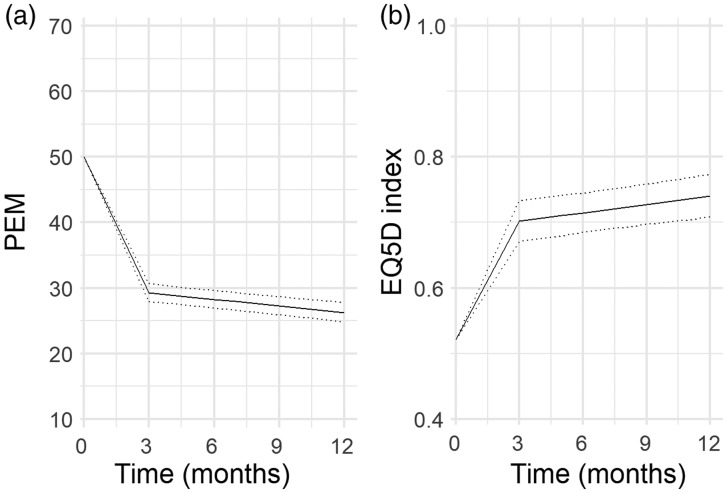
(a) The estimated trajectory of PEM part 2 score across time for all patients undergoing BTOA surgery (line represents mean expected score; dotted lines represent 95% confidence interval). (b) The estimated trajectory of EQ5D index score across time for all patients undergoing BTOA surgery (line represents mean expected score; dotted lines represent 95% confidence interval).

In the final model, improvement in both scores was seen, with the biggest improvement between baseline and 3 months (Figure 2). The regression model showed that patients who underwent LRTI had a slightly worse overall improvement in the PEM score (regression coefficient –0.40; 95% confidence interval –2.22 to –1.42), but this does not reflect a clinically meaningful difference (Supplementary Table 3). There was no difference between patients who underwent trapeziectomy alone or LRTI in the overall EQ5D score (regression coefficient –0.00, 95%CI –0.03 to 0.03).

## Discussion

This study shows that hand function and health state utility improve substantially after both trapeziectomy and trapeziectomy with LRTI in a UK population. In this cohort, there appears to be no meaningful difference in the change in PROMs postoperatively between the two techniques. This supports previous findings reported by other groups using different outcome measures (Wajon, 2015) and is in line with previous RCTs comparing the surgical techniques (Davis et al., 2004; De Smet et al., 2004; Field and Buchanan, 2007; Gangopadhyay et al., 2012). This study found larger postoperative improvements in the PEM than reported in the follow-up of the RCT reported by Salem and Davis (2014). Despite being a randomized trial, that smaller study also had similar attrition (54/114 thumbs were followed-up). The smaller PEM improvement seen by 6 years might represent ongoing decline in hand function in an ageing patient population after surgery. A mean improvement of 20 points, observed at 1 year following surgery, indicates a substantial improvement in hand function compared with the only identifiable interpretability estimate for the PEM, which is a minimal clinically important difference (MCID) estimated at 2.8–3 for Dupuytren’s disease (Dias, 2015).

Owing to the small numbers of patients undergoing procedures other than simple trapeziectomy or trapeziectomy with LRTI to date in the UKHR, only these procedures were included (Supplementary Table 4). Future work could compare outcomes following multiple techniques, including arthroplasty or arthrodesis, with trapeziectomy or LRTI and compare these results to previous clinical trials (Catalano et al., 2008; Kriegs-Au et al., 2005; Lovell et al., 1999; Nilsson et al., 2005; Schroder et al., 2002).

The EQ5D index is the preferred PROM for comparison of healthcare interventions with respect to patient-reported outcome and economic evaluation (NICE, 2012, 2013). The EQ5D index can range from –0.6 to 1, with ‘death’ at 0 and the best health state utility imaginable at 1. States worse than death result in negative values. A median improvement of 0.15 at 1 year following surgery indicates a significant improvement in the desirability of the state of quality of life. The improvement in the EQ5D index seen following BTOA surgery in this study is favourable to UK nationally reported PROMs for general surgical procedures and to many other widely commissioned musculoskeletal interventions (Jansson and Granath, 2011; Loveday et al., 2018; NHS Digital, 2019). The latest UK nationally reported PROMs quote an improvement of 0.45 for hip arthroplasty, 0.33 for knee arthroplasty, 0.081 for hernia repair, and 0.093 for varicose vein surgery.

This study uses observational data from routine clinical practice, which enables the results to be applied more generally. This is especially important as funding decisions become pragmatic (Makady et al., 2018), and this study adds to the evidence supporting the role of BTOA surgery within modern healthcare provision. There are few sources of routinely collected data worldwide that comprise significant proportions of both simple trapeziectomies to compare with LRTI, rather than a predilection towards one or the other. Therefore, this study is uncommon in being able to also compare the health utility of these two procedures.

This study has limitations. Entry into UKHR is currently voluntary and this could have led to selection bias of included patients. Comparison of patients undergoing surgery noted equally matched groups at baseline, but the observational nature of the data prevents further information surrounding surgical decision making for choosing one technique over another. It may be that trapeziectomy with LRTI is performed for specific indications that we cannot identify from the data available. Conversely, it may simply be that some surgeons prefer one technique over the other in general, in which case the comparison here is valid.

The UKHR does not include people who decline surgery for BTOA. As a result, it is impossible to confirm whether the changes seen after any type of surgery is true improvement from the surgery, and how much is attributable to regression to the mean (if people’s symptoms improved spontaneously) or response shift (if people’s perceptions changed over time – they accommodated to the state of their hand). Similarly, there may be confounders that could not be identified from the data available; for example, it is possible that postoperative rest improved other comorbidities and accounted for score improvement.

Over the first postoperative year there was a gradual reduction in completion rate of PROMs, which risks bias from loss to follow-up, though comparison of the groups with and without follow-up demonstrated similar characteristics. The attrition of PROM completion is commonly seen in surgical registries, with nationally collected PROMs in the UK consistently having a response rate of 56%–58% in hip arthroplasty, but only 23%–29% in hernia repair (NHS Digital, 2019). This is especially pertinent when one considers there is no financial incentive for surgeons or hospitals to participate in the UKHR, in contrast to the mandatory and incentivized collection of PROMs in UK hip and knee arthroplasty.

The use of the PEM as the hand-specific measure of outcome could also be considered a limitation by some, as there is a lack of evidence surrounding the interpretability of the PEM in BTOA (Rodrigues et al., 2015). In the context of this study, as there was improvement in the PEM of over 20 points from a score of 70, and a minimal important difference (MID) of 2.8–3 has been previously discussed in the context of Dupuytren’s contracture, we have considered this to represent a meaningful change for patients, as we are not aware of a published minimal important changes (MICs) for the PEM. Further work to establish MICs and MIDs for the PEM in BTOA would strengthen this interpretation of the data in this study. Our study suggests that BTOA surgery sits favourably among musculoskeletal surgery in its ability to improve health-related quality of life. As the study uses data taken from routine clinical practice, the generalizability of these results is clear for both clinicians and healthcare administrators.

## Supplemental Material

JHS909753 Supplemental Material1 - Supplemental material for Basal thumb osteoarthritis surgery improves health state utility irrespective of technique: a study of UK Hand Registry data Click here for additional data file. Supplemental material, JHS909753 Supplemental Material1 for Basal thumb osteoarthritis surgery improves health state utility irrespective of technique: a study of UK Hand Registry data by Jennifer C. E. Lane, Jeremy N. Rodrigues, Dominic Furniss, Edward Burn, Robert Poulter and Matthew D. Gardiner in Journal of Hand Surgery (European Volume)

JHS909753 Supplemental Material2 - Supplemental material for Basal thumb osteoarthritis surgery improves health state utility irrespective of technique: a study of UK Hand Registry data Click here for additional data file. Supplemental material, JHS909753 Supplemental Material2 for Basal thumb osteoarthritis surgery improves health state utility irrespective of technique: a study of UK Hand Registry data by Jennifer C. E. Lane, Jeremy N. Rodrigues, Dominic Furniss, Edward Burn, Robert Poulter and Matthew D. Gardiner in Journal of Hand Surgery (European Volume)

JHS909753 Supplemental Material3 - Supplemental material for Basal thumb osteoarthritis surgery improves health state utility irrespective of technique: a study of UK Hand Registry data Click here for additional data file. Supplemental material, JHS909753 Supplemental Material3 for Basal thumb osteoarthritis surgery improves health state utility irrespective of technique: a study of UK Hand Registry data by Jennifer C. E. Lane, Jeremy N. Rodrigues, Dominic Furniss, Edward Burn, Robert Poulter and Matthew D. Gardiner in Journal of Hand Surgery (European Volume)

JHS909753 Supplemental Material4 - Supplemental material for Basal thumb osteoarthritis surgery improves health state utility irrespective of technique: a study of UK Hand Registry data Click here for additional data file. Supplemental material, JHS909753 Supplemental Material4 for Basal thumb osteoarthritis surgery improves health state utility irrespective of technique: a study of UK Hand Registry data by Jennifer C. E. Lane, Jeremy N. Rodrigues, Dominic Furniss, Edward Burn, Robert Poulter and Matthew D. Gardiner in Journal of Hand Surgery (European Volume)

JHS909753 Supplemental Material5 - Supplemental material for Basal thumb osteoarthritis surgery improves health state utility irrespective of technique: a study of UK Hand Registry data Click here for additional data file. Supplemental material, JHS909753 Supplemental Material5 for Basal thumb osteoarthritis surgery improves health state utility irrespective of technique: a study of UK Hand Registry data by Jennifer C. E. Lane, Jeremy N. Rodrigues, Dominic Furniss, Edward Burn, Robert Poulter and Matthew D. Gardiner in Journal of Hand Surgery (European Volume)
