# Supplementary figures and images for: Basal thumb osteoarthritis surgery improves health state utility irrespective of technique: a study of UK Hand Registry data
Source: J Hand Surg Eur Vol. 2020 Mar 12;45(5):436–42. doi: 10.1177/1753193420909753 (PMC7232779; doi:10.1177/1753193420909753)

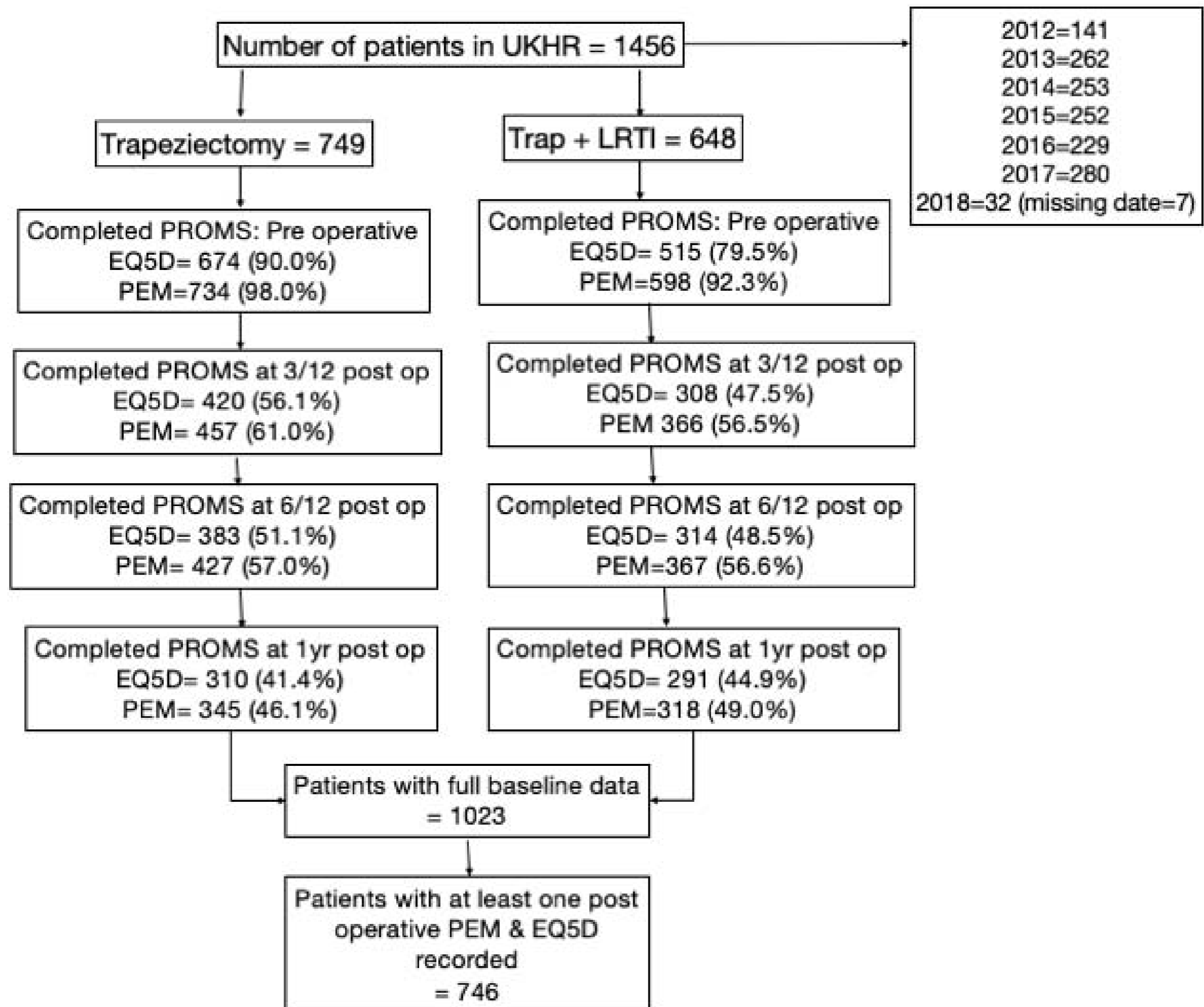

Supplement: JHS909753 Supplemental Material1 - Supplemental material for Basal thumb osteoarthritis surgery improves health state utility irrespective of technique: a study of UK Hand Registry data [file JHS909753_Supplemental_Material1.pdf]
