# Supplementary material for: Basal thumb osteoarthritis surgery improves health state utility irrespective of technique: a study of UK Hand Registry data
Source: J Hand Surg Eur Vol. 2020 Mar 12;45(5):436–42. doi: 10.1177/1753193420909753 (PMC7232779; doi:10.1177/1753193420909753)
Supplement: JHS909753 Supplemental Material2 - Supplemental material for Basal thumb osteoarthritis surgery improves health state utility irrespective of technique: a study of UK Hand Registry data [file JHS909753_Supplemental_Material2.pdf]

**Baseline Characteristics**	**Trapeziectomy (follow up)**	**Trapeziectomy (No follow up)**	**Trapeziectomy with LRTI (follow up)**	**Trapeziectomy with LRTI (No follow up)**
Median Age (IQR)	69<br>(63 to 73)	66<br>(60 to 72)	67<br>(61 to 72)	66<br>(58 to 71)
Sex	75.8% female	77.4% female	76.2% female	78.5% female
Median EQ5D index (IQR)	0.76<br>(0.46 to 0.80)	0.67<br>(0.26 to 0.80)	0.69<br>(0.26 to 0.80	0.65<br>(0.26 to 0.78)
Median PEM part 2 (IQR)	49<br>(41 to 56)	49<br>(40 to 57)	48<br>(39 to 55)	50<br>(42 to 56)

LRTI: Ligament reconstruction and Tendon Interposition
PEM: Patient Evaluation Measure
