# Supplementary material for: Basal thumb osteoarthritis surgery improves health state utility irrespective of technique: a study of UK Hand Registry data
Source: J Hand Surg Eur Vol. 2020 Mar 12;45(5):436–42. doi: 10.1177/1753193420909753 (PMC7232779; doi:10.1177/1753193420909753)
Supplement: JHS909753 Supplemental Material3 - Supplemental material for Basal thumb osteoarthritis surgery improves health state utility irrespective of technique: a study of UK Hand Registry data [file JHS909753_Supplemental_Material3.pdf]

		Regression analysis	Full dataset (Trapeziectomy)	Full dataset (Trapeziectomy with LRTI)
Number of patients included		746	749	648
Median Age (IQR)		67.0 (60.0 to 72.0)	67 (60 to 72)	66 (59 to 71)
Sex (%)	Female	78.8	77.1	78.1
Median Baseline PEM part 2 score (IQR)		50.0 (42.0 to 56.0)	49 (41 to 56)	49 (40 to 55)
Median baseline EQ5D index (IQR)		0.6 (0.2 to 0.8)	0.69 (0.26 to 0.80)	0.66 (0.26 to 0.78)

LRTI: Ligament reconstruction and Tendon Interposition
PEM: Patient Evaluation Measure
