# Supplementary material for: Basal thumb osteoarthritis surgery improves health state utility irrespective of technique: a study of UK Hand Registry data
Source: J Hand Surg Eur Vol. 2020 Mar 12;45(5):436–42. doi: 10.1177/1753193420909753 (PMC7232779; doi:10.1177/1753193420909753)
Supplement: JHS909753 Supplemental Material4 - Supplemental material for Basal thumb osteoarthritis surgery improves health state utility irrespective of technique: a study of UK Hand Registry data [file JHS909753_Supplemental_Material4.pdf]

	Post-operative PEM part 2 score		
Predictors	*Estimates*	*95% CI*	*p*
Baseline PEM part 2 score	0.31	0.23 – 0.40	<0.001
Sex: Male	2.55	0.31 – 4.80	0.026
Age	-0.22	-0.32 – -0.12	<0.001
Procedure: Trapeziectomy compared with LRTI	-0.40	-2.22 – 1.42	0.665
Time	-0.33	-0.45 – -0.21	<0.001
	**Post-operative EQ5D index**		
Predictors	*Estimates*	*95% CI*	*p*
bs(age, 3)2	0.59	0.26 – 0.92	<0.001
bs(age, 3)3	0.55	0.08 – 1.03	0.023
bs(eq5d_index_baseline, 3)1	1.22	0.94 – 1.50	<0.001
bs(eq5d_index_baseline, 3)3	1.05	0.88 – 1.22	<0.001
Procedure: Trapeziectomy compared with LRTI	-0.00	-0.03 – 0.03	0.806
sex: Male	-0.05	-0.09 – -0.01	0.010
bs(age, 3)1	0.73	0.03 – 1.43	0.042
bs(eq5d_index_baseline, 3)2	0.47	0.33 – 0.61	<0.001
time	0.00	0.00 – 0.01	<0.001

LRTI: Ligament reconstruction and Tendon Interposition
PEM: Patient Evaluation Measure
