# Supplementary material for: Basal thumb osteoarthritis surgery improves health state utility irrespective of technique: a study of UK Hand Registry data
Source: J Hand Surg Eur Vol. 2020 Mar 12;45(5):436–42. doi: 10.1177/1753193420909753 (PMC7232779; doi:10.1177/1753193420909753)
Supplement: JHS909753 Supplemental Material5 - Supplemental material for Basal thumb osteoarthritis surgery improves health state utility irrespective of technique: a study of UK Hand Registry data [file JHS909753_Supplemental_Material5.pdf]

**Procedure**	**Frequency**	**Percent**
Trapeziectomy	749	51.4
Trapeziectomy with LRTI	648	44.5
Total/Hemi arthroplasty	25	1.7
‘Revision’	16	1.1
Prosthetic spacer	7	0.5
CMC fusion	7	0.5
Prosthetic ligament stabilization	2	0.14
1st MC extension osteotomy	1	0.07
CMC stabilization	1	0.07
	1456	

LRTI: Ligament reconstruction and Tendon Interposition
CMC: carpometacarpal
MC: metacarpal
